# Gender-dependent associations between socioeconomic status and metabolic syndrome: a cross-sectional study in the adult Saudi population

**DOI:** 10.1186/1471-2261-14-51

**Published:** 2014-04-14

**Authors:** Nasser M Al-Daghri, Khalid M Alkharfy, Omar S Al-Attas, Nasiruddin Khan, Hanan A Alfawaz, Saad A Alghanim, Mansour A Al-Yousef, Abdulrahman S M Al-Ajlan, Majed S Alokail

**Affiliations:** 1 Center of Excellence in Biotechnology Research, King Saud University, Riyadh 11451, Kingdom of Saudi Arabia; 2 Biomarkers Research Program, Biochemistry Department, College of Science, King Saud University, PO Box, 2455, Riyadh 11451, Kingdom of Saudi Arabia; 3 Prince Mutaib Chair for Biomarkers of Osteoporosis, Biochemistry Department, College of Science, King Saud University, Riyadh 11451, Kingdom of Saudi Arabia; 4 Clinical Pharmacy Department, College of Pharmacy, King Saud University, Riyadh 11451, Kingdom of Saudi Arabia; 5 College of Food Science & Agriculture, Department of Food Science & Nutrition, King Saud University, Riyadh, Kingdom of Saudi Arabia; 6 Department of Health Administration, King Saud University, Riyadh, Kingdom of Saudi Arabia; 7 Health Affairs for Riyadh Region, Ministry of Health, Riyadh, Kingdom of Saudi Arabia; 8 Department of Clinical Lab Sciences, College of Applied Medical Sciences, King Saud University, Riyadh 11451, Kingdom of Saudi Arabia

**Keywords:** Gender, Socioeconomic status, Marital status, Income, Education, Saudi Arabia

## Abstract

**Background:**

To determine the gender-dependent association of socio-economic status variables with the prevalence of metabolic syndrome (MetS) in the adult Saudi population.

**Methods:**

A total of 9164 adult Saudis (aged 18–70 years) were included in this cross-sectional study. Marital status, income, education, and occupation were used as socio-economic indicators while behavioral factor like physical exercise was also taken into account. MetS was defined using the criteria based from the National Cholesterol Education Program Adult Treatment Panel III (NCEP-ATP III).

**Results:**

In males, the odds ratio (OR) of harboring MetS was higher in married [OR1.6 (Confidence Interval (CI) 1.1, 2.4); p < 0.03], and high income class [OR 2.3(CI 1.5, 3.5); p < 0.001] and lowest in retired and unemployed individuals [1.4(1.0, 1.9); p < 0.04, 0.61(0.45, 0.82); p < 0.001] respectively. In females, MetS was inversely related to high income [OR 0.70 (CI 0.46, 1.1); p < 0.09] and education level [OR 0.38 (CI 0.26, 0.56); p < 0.001], and was significantly higher in the unemployed class [OR 1.6 (CI 1.2, 2.2); p < 0.004].

**Conclusions:**

The prevalence of MetS is significantly high among retired, married and high-earning Saudi males while in females, high earners and high education seem to confer a protective effect against MetS.

## Background

Metabolic syndrome (MetS), considered to be a global epidemic [1], encompasses several risk factors including obesity, elevated arterial blood pressure, impaired glucose metabolism, and dyslipidemia. If it remains unchecked, it could lead towards higher susceptibility to type 2 diabetes, some cancers and cardiovascular disease [2-5]. The most widely used criteria for defining MetS is the NCEP ATP III definition [6] as it does not have a prerequisite risk factor as opposed to other definitions, favoring large scale screening for MetS assessment. MetS is influenced by various factors including diet, physical activity, gender, genetic background and age [7]. Moreover, the prevalence varies depending on the definition, as well as the composition of the population studied [8]. Worldwide, prevalence estimates for MetS in adult population varies from 8% in India [9] to 24.2% in United States [10] in men and from 7% in France [11] to 46.5% in India [12] in women. In Europe [13,14] and other Asian countries [15], the prevalence of MetS is higher in men than in women.

Based on the results of several studies and taking into consideration the different definitions, the prevalence of MetS in Gulf Cooperative Council (GCC) (Bahrain, Kuwait, Oman, Qatar, Saudi Arabia and the United Arab Emirates) countries varies from 20.7–37.2% [ATPIII definition [6] and 29.6–36.2% [International Diabetes Federation (IDF) definition [16] for males, and 32.1–42.7% (ATPIII definition) and 36.1–45.9% (IDF definition) for females [17,18]. However, in case of females, the prevalence rates of MetS in GCC states are almost 10–15% higher than in most developed countries [10,19].

The strong correlation between age and the prevalence of MetS has been established, as several studies have consistently demonstrated the high prevalence of MetS with advancing age [10,11]. A number of studies strongly link the socio-economic status with prevalence of MetS in different populations [20,21]. Although, the exact mechanism involved in this relationship is not clear, the socio-economic status may influence MetS and its components by affecting environmental and social factors [22,23]. Therefore, as an initial preventive approach to understand and manage the occurrence of MetS, it appears warranted to study the social and economic variables that could lead towards its high prevalence [24,25].

The rapid economic growth of Saudi Arabia has affected the behavior of the population in ways that is favorable for the manifestation of MetS [26]. In different age groups from different parts of Saudi Arabia, overweight and obesity are related with demographic, genetic and life style factors [27-30]. To date, there is limited information from Saudi Arabia in relation to cardiovascular diseases with demographic variables [31] and its possible link to MetS in males and females [32,33], respectively. To the best of our knowledge, no study specifically intended to relate the full MetS with socio-economic status variables based on gender difference with such a broad range of age groups.

The present study aims to assess the prevalence of full MetS based on NCEP ATP III diagnostic criteria and to examine its associations with socio-economic status variables (marital status, income, education and profession) within a broad age range (18–70 years) in adult Saudi males and females.

## Methods

### Participants and recruitment

A total of 9164 consenting Saudi adults [aged 18–70 years; 4417 males (mean age 41.35 ± 14.7) and 4747 females (37.4 ± 13.5)] were included in this cross-sectional study. The participants were part of the Biomarkers Screening Program Database, a collaboration between the Biomarkers Research Program (BRP) of King Saud University and the Ministry of Health in Riyadh, Kingdom of Saudi Arabia (RIYADH Cohort) done in March 1-August 31, 2009. Patient information was obtained from the database of more than 17,000 individuals. The information of individuals included in this study were based on inclusion criteria irrespective of disease status (diabetic, hypertensive and dyslipidemic patients were not excluded to avoid selection bias). Pregnant females were excluded from the study. Patients were recruited randomly from their homes using the cluster sampling strategy. They visited their nearest primary healthcare center (PHCC) that span the entire Riyadh region. The population of each PHCC was taken as a cluster, and the allocations of the required numbers of patients were proportional to the populations served by the PHCCs. No expatriates were included in the conduct of this study. Ethical approval was obtained from the Ethics Committee of the College of Science Research Center of King Saud University, Riyadh, Saudi Arabia [34].

### Anthropometric data and biochemical analyses

Subjects were requested to visit their respective PHCCs after overnight fasting (>10 hours) for anthropometry and blood withdrawal by the PHCC nurse and physician on duty, respectively. Anthropometry included height (rounded off to the nearest 0.5 cm), weight (rounded off to the nearest 0.1 kg), waist and hip circumference (centimeters), and mean systolic and diastolic arterial blood pressure (mmHg) (average of two readings after resting for 3–5 minutes). The body mass index (BMI) was calculated as weight in kilograms divided by height in squared meters. A fasting blood sample was collected and transferred immediately to a non-heparinized tube for centrifugation. Collected serum was then transferred into a pre-labeled plain tube, stored in ice and delivered to the Biomarkers Research Program (BRP) laboratory of King Saud University, Riyadh, Saudi Arabia, on the same day of collection for immediate storage at −20°C freezer pending further analysis. The blood samples were analyzed for fasting glucose and lipid profile including HDL-C, LDL-C, and triglycerides using a chemical analyzer (Konelab, Espoo, Finland).

Participants completed an interviewer-administered questionnaire covering information on socioeconomic status, physical exercise, personal medical history, family history, and current drug therapy. Socio-economic status was assessed based on the questionnaire that included marital status, monthly income, occupation, and education. Marital status was recorded in four categories: single, married, divorced, and widowed. Total monthly household income was divided in categories [depending on the amount of Saudi Arabian Riyal (1SAR = 0.266USD): upper income class (10,000-20,000), middle income class per month (>5000 – 10,000); and low income class (< 5000). The classification of occupation was based on current employment status and sectors, as government, private, retired and unemployed (non-officially employed). Education level was categorized as: uneducated (< 6 years), precollege (7–12 years), and college/high education (>12 years). Physical activity was divided into five groups according to frequency of exercise: daily, three or four times per week, one or two three times per week, few times a month, and once a month.

### Statistical analyses

Statistical analysis was performed using SPSS version 16.0 (SPSS, Chicago, IL, USA). Demographic and biochemical characteristics of the study population, were compared using the Chi-square or Fisher’s exact test for categorical variables. Evaluation of trend between age groups was analyzed using the Cochran-Armitage trend test. Odds ratios (ORs) and 95% confidence intervals (95% CIs) for metabolic syndrome with various socio-economic parameters were calculated using multivariate logistic regression analysis adjusted for age. P values less than 0.05 were accepted to indicate statistically significant differences.

### Metabolic syndrome definition

All subjects were screened for MetS. Definition of MetS and its components were based on the ATPIII Guidelines [6], by the presence of three or more of the following risk factors: central obesity (Waist circumference ≥90 cm for men and ≥80 cm for women); systolic blood pressure ≥130 mmHg and/or diastolic blood pressure ≥85 mmHg; fasting glucose ≥100 mg/dL; TG ≥150 mg/dL; HDL-C levels (< 40 mg/dL for men and < 50 mg/dL for women). In addition, subjects who were on medications taking antihypertensive or antidiabetic drugs were considered to have elevated BP or high fasting glucose levels, respectively.

## Results

The overall prevalence of MetS and its individual components based on demographic and socioeconomic status variables are presented in Table 1, following ATPIII criteria [6].

**Table 1:** Overall prevalence of MetS according to demographic and socio-economic status characteristics based on ATPIII Criteria

	**Waist Circumference (Men > 102, Women >88 cm), n (%)**	**Triglycerides > 1.7 mmol/l, n (%)**	**FBG (≥6.1 mmol/l), n (%)**	**Hypertension (≥130/85 mmHg), n (%)**	**HDL (Men < 1.03 mmol/l); (Women < 1.29 mmol/l), n (%)**	**MetS (ATPIII), n (%)**
Male	1480 (33.5)*	2200 (49.8)*	1829 (41.4)*	1572 (35.6)*	3781 (85.6)*	2085 (47.2)*
Female	2606 (54.9)	1281 (27.0)	1571 (33.1)	1234 (26.0)	3798 (80.0)	1913 (40.3)
**Marital status**						
Single	409 (18.0)	439 (19.3)	330 (14.5)	222 (9.7)	1695 (74.6)	296 (13.0)
Married	3322 (51.9)	2823 (44.1)	2800 (43.7)	2338 (36.5)	5481 (85.5)	3374 (52.7)
Divorced	101 (73.5)	56 (40.8)	47 (34.7)	42 (30.6)	120 (87.8)	70 (51.0)
Widowed	254 (73.4)*	163 (46.9)*	223 (64.1)*	204 (58.6)*	283 (81.2)*	258 (74.2)*
**Income**						
Low income	2075 (48.7)	1180 (27.7)	1334 (31.3)	1090 (25.6)	3409 (80.0)	1612 (37.8)
Middle Class	1182 (40.7)	1354 (46.6)	1231 (42.4)	953 (32.8)	2491 (85.7)	1409 (48.5)
Upper Class	829 (41.5)*	947 (47.4)*	835 (41.8)*	763 (38.2)*	1679 (84.0)*	977 (48.9)*
**Education**						
Uneducated (< 6 yrs)	1697 (66.8)	1071 (42.2)	1324 (52.3)	1172 (46.2)	2121 (83.6)	1594 (62.8)
Pre-college (7–12 yrs)	1701 (39.4)	1740 (40.3)	1553 (36.1)	1207 (28.0)	3629 (84.2)	1798 (41.8)
Higher Educated (>12 yrs)	688 (29.7)*	670 (28.9)*	523 (22.6)*	427 (18.4)*	1829 (78.9)*	606 (26.1)*
**Profession**						
Government	1172 (40.0)	1350 (46.0)	1075 (36.8)	850 (29.0)	2451 (83.6)	1260 (43.0)
Private	150 (34.1)	215 (48.8)	188 (42.7)	147 (33.5)	405 (92.1)	228 (51.8)
Retired	382 (44.4)	490 (56.9)	546 (63.4)	457 (53.1)	764 (88.8)	592 (68.6)
Unemployed	2382 (48.3)*	1426 (28.9)*	1591 (32.3)*	1352 (27.4) *	3959 (80.3)*	1918 (38.8)*
**Exercise**						
Daily or 3–4 times/week	2813 (43.8)	2215 (34.5)	2279 (35.5)	1716 (26.7)	5160 (80.2)	2733 (42.5)
1-2 times/week	794 (46.7)	800 (47.0)	684 (40.2)	598 (35.2)	1495 (87.9)	764 (44.9)
Few times/Once a month	479 (46.3)*	466 (45.1)*	437 (42.2)*	492 (47.6)*	924 (88.6)*	501 (48.3)*

*p < 0.05: comparison between various classes of demographic and socioeconomic status characteristics.

As regards the marital status, the widow class presented higher prevalence of metabolic syndrome (74.2%) as compared to other classes (single, married and divorced). A significant inverse relationship of income with prevalence of MetS was found, being highest in the upper class (48.9%) than other classes. Level of education had an inverse relation, with the lowest prevalence of MetS in higher education level (26.1%) than uneducated and pre-college subjects. Based on occupation, the officially unemployed participants presented the lowest (38.8%) prevalence of MetS than other professional classes. Physical exercise showed an inverse association with the prevalence of MetS, being highest in the category with ‘few times a month/once a month’ group (48.3%) than other groups.

**Figure 1:**
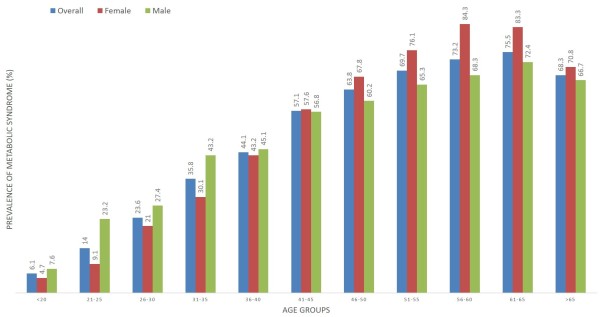
**Prevalence of MetS based on quartiles of age and gender, according to ATPIII criteria.** The overall prevalence of MetS increased uniformly with increase in age in both males and females. In males, a direct relationship between age and the full MetS was found in the range of 20–40 years which was lost in later age groups. However, in females, there was an increase in the prevalence of MetS from age 41 to 70 years. A decrease in the prevalence of MetS was observed in the age group of >65 years.

The pattern of distribution regarding prevalence of MetS (%) based on age groups and sex is presented in Figure 1. The overall prevalence of MetS increased uniformly with increase in age in both males and females. With age, the prevalence of MetS increased from 9.1 among females of age group < 20 yrs to 70.8 in >65 yrs, representing a significant trend (p < 0.001). Similar results were observed in males with a significant increase from 7.6 to 66.7 (p < 0.001). Table 2 shows a significant gender difference among various socioeconomic variables and behavioral factor. The prevalence of MetS was 47.2% for males and 40.3% for females.

**Table 2:** Gender difference among socioeconomic variables and behavioral factor

	**Males**	**Females**	**p value**
**N**	**4417**	**4747**	
**Marital Status (%)**			0.008
Single	22.5	26.9	
Married	77.2	63.2	
Divorced	0.1	2.7	
Widowed	0.2	7.2	
**Income (%)**			
Low income	16.1	74.9	< 0.001
Middle Class	48.2	16.3	
Upper Class	35.7	8.8	
**Education (%)**			
Uneducated (< 6 yrs)	16.9	38.0	0.003
Pre college (7–12 yrs)	56.0	38.3	
Higher Education (>12 yrs)	27.1	23.7	
**Profession (%)**			
Government	50.7	14.5	< 0.001
Private	8.8	1.2	
Retired	18.7	0.7	
Unemployed	21.8	83.6	
**Exercise (%)**			
Daily or 3–4 times/week	57.7	80.8	0.002
1-2 times/week	26.2	12.2	
Few times/Once a month	16.1	7.0	
**Mets (%)**	47.2	40.3	< 0.001

p-value significant at < 0.05.

Table 3 presents the odds ratios [ORs (95% CI)] indicating the prevalence of having the full MetS with different socio-economic status variables in males. After adjustment for age, a significant relation showing high prevalence of MetS within the married class was observed [1.6 (1.1, 2.4); p < 0.03]. The income group revealed a higher prevalence in the middle as well as upper income classes [2.2 (1.5, 3.5); p < 0.001 and [2.3 (1.5, 3.5); p < 0.001], respectively, than low income subjects. The retired and officially unemployed males showed the highest and lowest odds for MetS [1.4(1.0, 1.9); p < 0.04, 0.61(0.45, 0.82); p < 0.001] respectively. Self-assessed physical exercise was not significantly influencing MetS in male participants.

**Table 3:** Prevalence of metabolic syndrome according to socio-economic variables, in males

**Marital status**	**Odds ratio**^**a**^	**P value**	**Odds ratio**^**b**^	**p value**
	**(95% CI)**		**(95% CI)**	
Single	1.0		1.0	
Married	6.3 (4.6, 8.5)	< 0.001	1.6 (1.1, 2.4)	0.03
Divorced	4.9 (0.30, 80.2)	0.26	0.96 (0.05, 16.8)	0.98
Widowed	9.9 (0.88, 110.9)	0.06	1.9 (0.15, 22.6)	0.61
**Income**				
Low Income	1.0		1.0	
Middle Class	6.1 (4.2, 8.7)	< 0.001	2.2 (1.5, 3.5)	< 0.001
Upper Class	6.3 (4.3, 9.1)	< 0.001	2.3 (1.5, 3.5)	< 0.001
**Profession**				
Government	1.0		1.0	
Private	1.5 (1.1, 2.1)	0.02	1.4 (0.97, 2.1)	0.06
Retired	2.6 (2.0, 3.5)	< 0.001	1.4 (1.0, 1.9)	0.04
Unemployed	0.39 (0.30, 0.52)	< 0.001	0.61 (0.45, 0.82)	0.001
**Education**				
Uneducated (< 6 yrs)	1.0		1.0	
Precollege (7–12 yrs)	0.61 (0.46, 0.81)	0.001	1.2 (0.94, 1.8)	0.10
Higher Education (>12 yrs)	0.40 (0.29, 0.54)	< 0.001	1.0 (0.70, 1.4)	0.95
**Exercise**				
Daily or 3–4 times/week	1.0		1.0	
1-2 times/week	1.2 (0.92, 1.6)	0.15	1.3 (0.97, 1.8)	0.06
Few times/Once a month	1.2 (0.88, 1.7)	0.22	1.3 (0.92, 1.9)	0.12

^a^- Crude odds ratio; ^b^- Age-adjusted; p-value significant at < 0.05.

The odds ratios for the prevalence of having the full MetS in females is presented in Table 4. The prevalence of MetS was significantly higher among unemployed women [1.6 (1.2, 2.2); p < 0.004] and significantly lower in middle, upper income class and higher education level [0.65 (0.48, 0.89); p < 0.008, 0.70 (0.46, 1.1; p < 0.09)] and [0.38 (0.26, 0.56); p < 0.001] respectively.

**Table 4:** Prevalence of metabolic syndrome according to socio-economic variables in females

**Marital status**	**Odds ratio**^**a**^	**P value**	**Odds ratio**^**b**^	**p value**
	**(95% CI)**		**(95% CI)**	
Single	1.0		1.0	
Married	8.4 (6.1, 11.6)	< 0.001	1.3 (0.85, 2.1)	0.21
Divorced	9.1 (4.7, 17.4)	< 0.001	1.2 (0.58, 2.6)	0.56
Widowed	25.4 (15.4, 41.9)	< 0.001	1.5 (0.77, 2.8)	0.23
Income				
Low income	1.0		1.0	
Middle class	0.81 (0.62, 1.1)	0.12	0.65 (0.48, 0.89)	0.008
Upper class	0.64 (0.45, 0.92)	0.01	0.70 (0.46, 1.1)	0.09
Profession				
Government	1.0		1.0	
Private	0.50 (0.16, 1.5)	0.22	1.0 (0.28, 3.6)	0.98
Retired	1.8 (0.59, 5.6)	0.29	0.77 (0.22, 2.6)	0.68
Unemployed	1.5 (1.2, 2.1)	0.003	1.6 (1.2, 2.2)	0.004
Education				
Uneducated (< 6 yrs)	1.0		1.0	
Precollege (7–12 yrs)	0.28 (0.23, 0.36)	< 0.001	0.82 (0.62, 1.1)	0.17
Higher education (>12 yrs)	0.09 (0.07, 0.13)	< 0.001	0.38 (0.26, 0.56)	< 0.001
Exercise				
Daily or 3–4 times/week	1.0		1.0	
1-2 times/week	1.3 (0.94, 1.8)	0.10	1.3 (0.91, 1.9)	0.13
Few times/Once a month	0.71 (0.45, 1.1)	0.15	0.67 (0.39, 1.1)	0.13

^a^-Crude odds ratio; ^b^ – Age adjusted. p-value significant at < 0.05.

## Discussion

The results of this study, one of the largest population-based studies regarding MetS in Saudi Arabia, demonstrate the high prevalence of MetS in both adult males than females. The results showed a significant positive relationship between married status and high income class in males and a higher prevalence of MetS. Moreover, officially unemployed males showed the lowest prevalence of having the full MetS. In females, those with no professional activities were more predisposed to develop MetS while the higher education and high income level seem to confer a protective effect against MetS.

The present results, with an increased prevalence of MetS with advancing age in both genders, support several other studies performed in different populations [7,35,36]. The over-all prevalence of MetS in males was higher only in the age group of 40 years and below as compared to females, probably due to the presence of high number of younger subjects in this age group. However, the females in the age group of 45–60 years emerged as the highest contributor in the prevalence of MetS as compared to males. The present results supports the study performed by Al-Daghri and colleagues in urban Saudi adults, demonstrating higher prevalence of MetS in males only in the younger age groups (40 years and below) which is eventually replaced by females in the older age groups (49–55 years) [37]. In the present study, a decrease in the prevalence of MetS in the age group >65 years was noted in both males and females. The most probable reason for this low prevalence is the lower number of individuals belonging to this age group.

The full MetS is associated significantly with marital status in adults (20-65 yrs) as shown by Sirdah et al. [38]. Studies in females from Iran [39] and Morocco [40] demonstrated a similar trend. The present study partially supports the above studies showing higher prevalence of MetS in married couples but only in males as compared to other classes.

Gender-related differences in MetS are a matter of controversy with no relation to income or education level in males and females [41], a positive association of MetS with higher education and income among males but not in females [42], or an inverse association of income with MetS only in females and educational level associated with MetS in both genders [20]. The higher socioeconomic groups in Tunisian adults demonstrated higher MetS rates [43]. In addition*,* Park and colleagues observed the higher prevalence of MetS in males with highest household income [44]. Ferguson et al. [42] demonstrated the association of high income with increased odds of having the MetS among males, adjusted for age-group. However, these findings contradicts the results for developed countries, where lower income is associated with higher prevalence of MetS [45,46]. Depending on the developmental level of specific countries [47], it has been shown that in developed countries, CVD risk factor burden are inversely related to socioeconomic status while a positive association exists in less developed or low and middle income countries [47,48]. The present results are in agreement with the findings of Ferguson and Park et al. [42,44] showing a positive association between higher income and the prevalence of metabolic syndrome in males but showed no relation of education with such prevalence. The rapidly expanding economic status of Saudi Arabia has affected its cultural lifestyle during the last few decades, pushing towards physical inactivity and sedentary behavior, a leading cause for increasing rate of obesity [49] that could also be an important contributing factor towards higher prevalence of MetS. The fast economic growth in Saudi Arabia diverted the general population towards more dependency on automobile, telecommunication technology, use of high fat and dense-caloric foods, and decreased occupational-work demands. These factors could contribute to minimize requirement for physical activity and add more sedentary lifestyles [50]. In addition, the habit of eating out has been highly prevalent in most middle-east countries including Saudi Arabia, because of an increase in income per capita among these people [51].

Based on religion and cultural aspects, the males in Saudi Arabia had much more accessibility and opportunity to eat out more frequently than females and therefore pay less attention towards healthy food choices. It has also been shown that the purchasing power of males with increase in earnings is not balanced for the adaptation of a healthier lifestyle [52,53]. Therefore, in present study, a positive association between prevalence of MetS and higher income class in males could be due to sedentary lifestyle, lack of proper food choices and the increased ability to purchase foods as compared to females belonging to the same income status.

Furthermore, males in the lower income class seem to be involved with more physically demanding occupations and hence present a lower prevalence of metabolic syndrome than females. In the present study, the retired and officially unemployed males presented the highest and lowest prevalence of MetS respectively. As explained earlier, the unemployed group may mostly consist of males involved in more physically demanding activities which may prove beneficial against developing MetS, as compared to working in administrative or academic fields.

As compared to males, there was no significant association between age-adjusted marital status and prevalence of MetS in females. In a recent study, the education level was demonstrated as an important socioeconomic determinant of MetS only in females [54]. There are studies demonstrating a less prominent inverse association between educational level and MetS in males as compared to females [24,55]. Zhan and colleague’s demonstrated high risk of MetS among females with lower education and household monthly income level, while such association was not significant in males [56]. Similar results were observed in an urban population in Kenya showing an inverse association of the presence of MetS with attainment of higher education in females but not in males [57]. Moreover, the inverse association of income and education with prevalence of MetS in females has been documented in several studies [20,39,58,59]. The present results support the above findings showing a strong, significant inverse relation between high income and education with prevalence of MetS. The reason for this association in females may be related to the positive and favorable effect of education in preferring adequate food sources [22,60] and adopting healthy behaviors [61,62] that could in turn lead to lower prevalence of MetS. Moreover, females are more health conscious than males and use their knowledge to acquire healthier lifestyle patterns. In the present study, the OR for MetS increased in the unemployed female category. Since, these are mostly housewives with almost negligible involvements in any type of physical exercise, such sedentary behaviors are a plausible cause for the high prevalence of metabolic syndrome [63]. In Saudi Arabia, females have fewer chances to move outside their homes and have limited opportunities to attend health centers [64]. Saudi Arabian culture does not allow females to undertake outdoor physical activities, and related facilities for females are lacking. In addition, the hot climate, use of automobiles even for shorter distances, and hiring of domestic helpers, seems to contribute to physical inactivity in daily life [65].

As in males, no significant association was observed between physical exercise and prevalence of metabolic syndrome in females. There are some limitations while interpreting the results of this study. Firstly, the socio-economic status variables like household income and education are interrelated and thus it is not easy to demonstrate the individual participation and association of these variables with MetS and its prevalence. Being a cross sectional study, the results are unable to show a causal relationship between MetS and the socio-economic status.

## Conclusion

Gender influenced the socio-economic status indicators and its association with the prevalence of MetS. The prevalence was more frequent in males belonging to married, high income and retired individuals. On the contrary, higher education and income played an important role in decreasing the prevalence of syndrome in females, with no such education gradient in males. Apart from health awareness education programs for proper life style behavior particularly in males, it is recommended that the jobless and retired individuals (males and females) should indulge in some sort of physical activity and be educated for proper food choices, as preliminary precautions in order to decrease the high prevalence of metabolic syndrome. Preventive strategies and knowledge regarding modifiable risk factors must be taken into account in both adult males and females.

## Abbreviations

NCEP ATP III: National Cholesterol Education Program Adult Treatment Panel III; IDF: International Diabetes Federation; GCC: Gulf Cooperative Council; PHCC: Primary healthcare center; HDL-C: High density lipoprotein-cholesterol; LDL-C: Low density lipoprotein-cholesterol; TG: Triglycerides; FBG: Fasting blood glucose; SAR: Saudi Arabian Riyal; USD: United States Dollar.

## Competing interests

The authors declare that they have no competing interests.

## Authors’ contributions

NMA, KMA, and OSA contributed in the design, subject recruitments and data collection. NK, HAA, ASMA and SAA carried out sample analysis, interpretation, and preparation of draft manuscript. ASMA, MAA, and MSA edited the final version of the manuscript. All authors read and approved the final manuscript.

## Pre-publication history

The pre-publication history for this paper can be accessed here:

http://www.biomedcentral.com/1471-2261/14/51/prepub
